# Protein labeling for live cell fluorescence microscopy with a highly photostable renewable signal†
†Electronic supplementary information (ESI) available: Supplementary methods, figures, movies, and data. See DOI: 10.1039/c7sc01628j


**DOI:** 10.1039/c7sc01628j

**Published:** 2017-08-03

**Authors:** Nina G. Bozhanova, Mikhail S. Baranov, Natalia V. Klementieva, Karen S. Sarkisyan, Alexey S. Gavrikov, Ilia V. Yampolsky, Elena V. Zagaynova, Sergey A. Lukyanov, Konstantin A. Lukyanov, Alexander S. Mishin

**Affiliations:** a Shemyakin-Ovchinnikov Institute of Bioorganic Chemistry , Moscow , Russia . Email: mishin@ibch.ru; b Nizhny Novgorod State Medical Academy , Nizhny Novgorod , Russia; c Centre for Genomic Regulation (CRG) , The Barcelona Institute for Science and Technology , Dr Aiguader 88 , 08003 Barcelona , Spain; d Pirogov Russian National Research Medical University , Moscow , Russia

## Abstract

We present protein-PAINT – the implementation of the general principles of PAINT (Point Accumulation for Imaging in Nanoscale Topography) for live-cell protein labeling. Our method employs the specific binding of cell-permeable fluorogenic dyes to genetically encoded protein tags. We engineered three mutants of the bacterial lipocalin Blc that possess different affinities to a fluorogenic dye and exhibit a strong increase in fluorescence intensity upon binding. This allows for rapid labeling and washout of intracellular targets on a time scale from seconds to a few minutes. We demonstrate an order of magnitude higher photostability of the fluorescence signal in comparison with spectrally similar fluorescent proteins. Protein-PAINT ensures prolonged super-resolution fluorescence microscopy of living cells in both single molecule detection and stimulated emission depletion regimes.

## Abstract 2

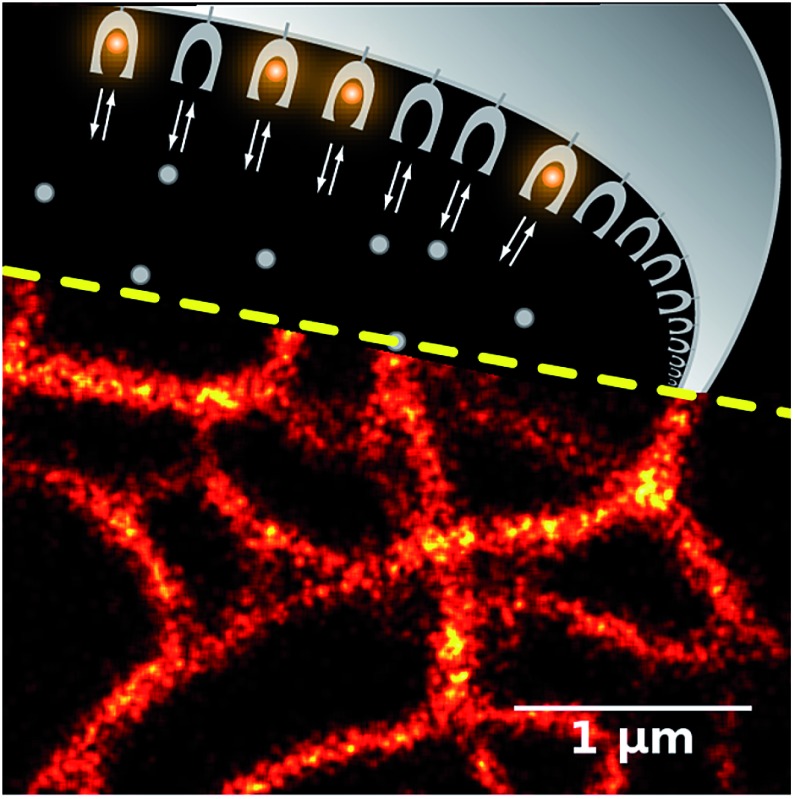
A novel method of protein labeling uses the highly dynamic reversible association of a cell-permeable fluorogenic dye and lipocalin Blc mutants.

## Introduction

Fluorescence labeling of target proteins in live cells provides opportunities to visualize their intracellular distribution, interactions, and dynamics. Green Fluorescent Protein (GFP) and GFP-like proteins are most often used for this purpose as fully genetically encoded labels.^1^ Also, methods of protein labeling based on protein tags that covalently bind chemical fluorophores have been developed.^2^–^7^ In comparison to labeling with fluorescent proteins, these methods provide more flexibility in choosing the spectral characteristics of the dyes and the time of labeling, but require additional steps of prolonged incubation and washing out the excess of unbound dye.

These steps could be avoided by using fluorogenic dyes – those that are non-fluorescent in solvents but become fluorescent in a complex with the molecule of interest.^8^–^13^ The first system for specific protein labeling using exogenous synthetic fluorogens consisted of single-chain antibodies (FAPs) against thiazole orange or malachite green.^14^ More recently, the Y-FAST^15^ protein was engineered on the basis of the blue-light photoreceptor from *Halorhodospira halophila* to reversibly bind fluorogenic rhodanine derivatives. However, both labeling systems do not provide an increase in photostability, which could be expected from the exchange of the protein-bound and free dye molecules from solution.

Constant exchange of the dye provides a method for efficient single molecule super-resolution microscopy, since the binding is detected as a flash of fluorescence signal. The original demonstration of this principle on membranes with the Points Accumulation for Imaging in Nanoscale Topography (PAINT) technique^13^ was further extended to the labeling of arbitrary targets with pairs of DNA strands^16^ (DNA-PAINT), one of which is fluorescent and the other one is conjugated to the target or binder (*e.g.* antibody). However, even the latest-generation^17^,^18^ multiplexed DNA-PAINT variants are still limited by design to fixed samples.

Here, we created novel fluorogen–protein pairs for live-cell protein labeling and validated them in widefield, confocal and super-resolution microscopy applications. With these pairs we demonstrate the protein-PAINT principle: the combination of the genetically encoded protein tag and the rapid exchange of the fluorogenic dye allows for on-demand fluorescent labeling of target proteins in living cells and is compatible with various super-resolution techniques.

## Results and discussion

GFP chromophore analogs have been previously reported to be fluorogenic with RNA aptamers^10^ and protein hosts.^19^–^21^ Many of them combine the ability to penetrate cell membranes with low brightness in the unbound state, and can dramatically increase the quantum yield when conformationally locked in a pocket of a host macromolecule.

Application of a chromophore-containing solution to cells expressing host proteins or RNA allows for the fast formation of fluorescent complexes, but also – if the dye is not bound to the host too tightly – for efficient exchange of dye molecules with the pool of free molecules. Therefore, photobleached dye molecules can be quickly exchanged for the fluorescent ones. Taking this as a starting point, we designed and synthesized the library of GFP chromophore analogs, GFP-like aminated chromophores, and conformationally locked compounds^22^ (see ESI Methods†). For the protein counterpart we have chosen the lipocalin family of proteins as a scaffold capable of reversibly binding various small-molecule ligands.^23^–^26^ To avoid undesirable specific protein–protein interactions in eukaryotic systems we focused on bacterial proteins and selected the monomeric lipocalin Blc from *E. coli* without intramolecular disulphide bonds.^27^

**Fig. 1:**
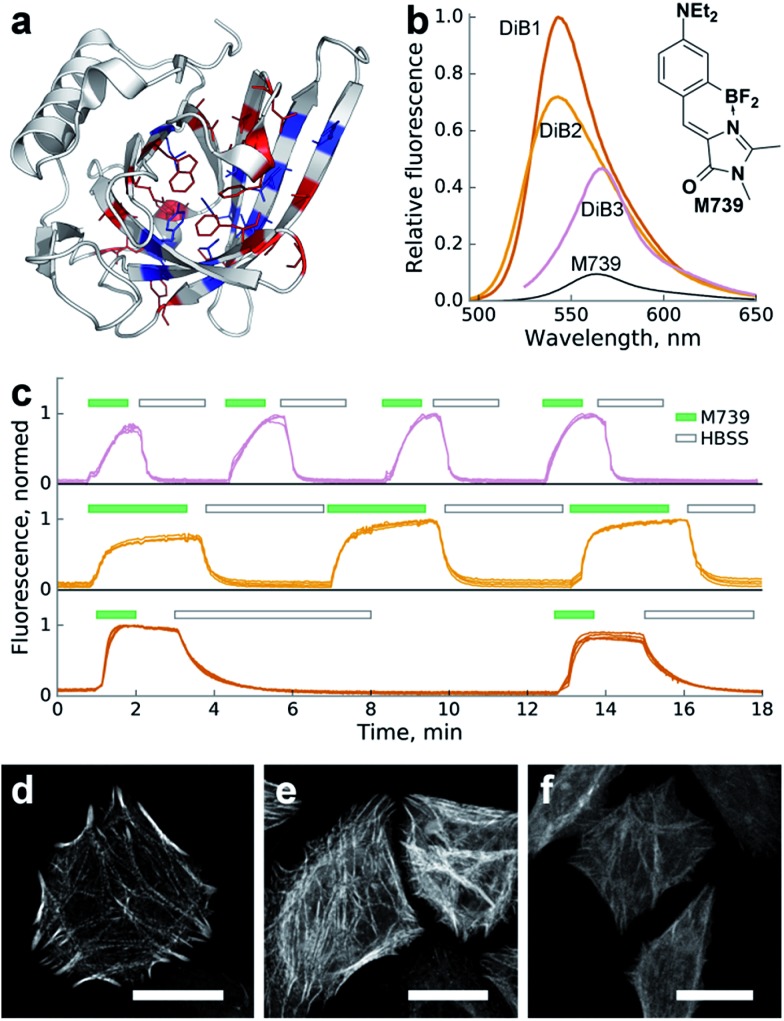
Characterization of the Blc mutants in a complex with chromophore **M739***in vitro* and *in cellulo*. (a) Locations of the amino acid sites of Blc analyzed in this work, highlighted in the wild-type crystal structure (PDB ID: ; 1QWD). Pocket-facing amino acid positions are colored. Sites with mutations tested *in vitro* are shown in blue. (b) Changes in fluorescence upon complex formation. Shown are emission spectra of the same concentration of **M739** (free or in the presence of saturating amounts of the corresponding proteins) normalized for the **DiB1** maximal value. (c) Sequential staining and washout of H2B with **DiB**s in live HeLa cells. Top-to-bottom: **DiB3**, **DiB2** and **DiB1**. The green filled and black hollow rectangles above the curves designate addition of the **M739** solution (0.5 μM) or washout with HBSS buffer, respectively. Multiple intensity profiles correspond to different cells; on-to-off signal ratios are approximately 30, 15, and 30 for **DiB3**, **DiB2**, and **DiB1**, respectively. (d–f) Confocal fluorescence microscopy of **DiB**s in a HeLa cell line; (d) α-actinin-**DIB1** in the presence of 0.25 μM **M739** (excitation: 488 nm, emission: 520–560 nm); (e) α-actinin-**DIB2** in the presence of 1 μM **M739** (excitation: 488 nm, emission: 520–560 nm); (f) α-actinin-**DIB3** in the presence of 5 μM **M739** (excitation: 543 nm, emission: 560–600 nm). Scale bars – 20 μm.

We performed *in silico* mutagenesis of amino acids within the ligand-binding pocket of Blc (Fig. 1a) and then computationally screened the generated library using molecular docking of GFP chromophores. Mutants with an incompatible pocket size were filtered out as previously described.^21^ As a result, we selected nineteen top-scoring mutants (Fig. S1†) for cloning, expression and screening *in vitro* against the chromophore library.

We observed an increase in fluorescence intensity in a number of protein–chromophore pairs (Fig. S2†). We excluded chromophores that showed too broad cross-reactivity with mutant proteins (*e.g.***1167** in Fig. S2†) from further characterisation. We also filtered out proteins with intense visible coloring or autofluorescence *in vitro* or in cell culture (Fig. S2,† right column).

For further characterisation we selected the three best-performing complexes of proteins with chromophore **M739** that possessed different dissociation constants and called them **DiB1** (“Dye in Blc 1”, *K*_d_ = 0.1 μM), **DiB2** (*K*_d_ = 4 μM) and **DiB3** (*K*_d_ = 9 μM) (Tables 1 and S1†). Within the protein complexes, **M739** showed blue- (for **DiB1** and **DiB2**) or red-shifted (for **DiB3**) absorption spectra with similar extinction coefficients in the range of 43 000–51 000 M^–1^ cm^–1^ (Fig. S3,† Table 1). Compared to free **M739**, these complexes possessed an ∼5–10-fold increase in the fluorescence quantum yield.

**Table 1:** Properties of selected M739-based **DiB**s

Pair name	Mutations^*a*^	*λ*_ex_, nm	*λ*_em_, nm	FQY^*b*^, %	EC, M^–1^ cm^–1^	*λ*-Dependent FI, fold^*c*^	*K*_d_, μM	Photostability (relative to)^*d*^
**DiB1**	A36C/L141N	513	542	32	45 800	52	0.1	2× (EGFP)
**DiB2**	A36C	510	539	32	51 000	64	4	6× (EGFP)
**DiB3**	V74F/L141Q	546	565	15	43 000	11	9	10× (mKate)
Free **M739**	N/A	520	563	3.5	53 500	N/A		

^*a*^For numbering and sequence information refer to ESI Fig. 1.†

^*b*^Fluorescence quantum yield.

^*c*^Fluorescence increase compared to free **M739** under conditions of confocal or TIRF microscopy (excitation at 488 nm and detection at 500–530 nm for **DiB1** and **DiB2**; excitation at 561 nm and detection at 580–620 nm for **DiB3**).

^*d*^See Fig. 2.

But since the binding also led to a blue-shift of the emission spectra of **DiB1** and **DiB2**, the effective wavelength-dependent fluorescence increase of the complex was more than 50-fold (Fig. 1b and Table 1).

To test the **DiB**s' performance in mammalian cells we expressed them as fusions with various proteins of interest (histone H2B, cytokeratin, α-actinin and vimentin). We found that the two yellow-colored pairs (**DiB1** and **DiB2**) performed well in both widefield and confocal fluorescence microscopy. The target intracellular structures of the live cells demonstrated bright fluorescence immediately upon addition of the fluorogen **M739** at concentrations of 0.1–1 μM (Fig. 1d and e and S4†). Thanks to the cell-permeable **M739** fluorogen, multiple staining and washout cycles could be repeated in rapid succession with the cell-perfusion system (Fig. 1c), which is not possible with labeling systems with ester-modified fluorogens.^28^ Likely due to the lower affinity of the complex, labeling with **DiB3** required a higher fluorogen concentration, which resulted in a higher level of background fluorescence (Fig. S5†). Therefore, **DiB3** was found to have a limited utility for widefield microscopy, while **DiB1** and **DiB2** performed almost as well as a fluorescent protein (Fig. S5†).

**Fig. 2:**
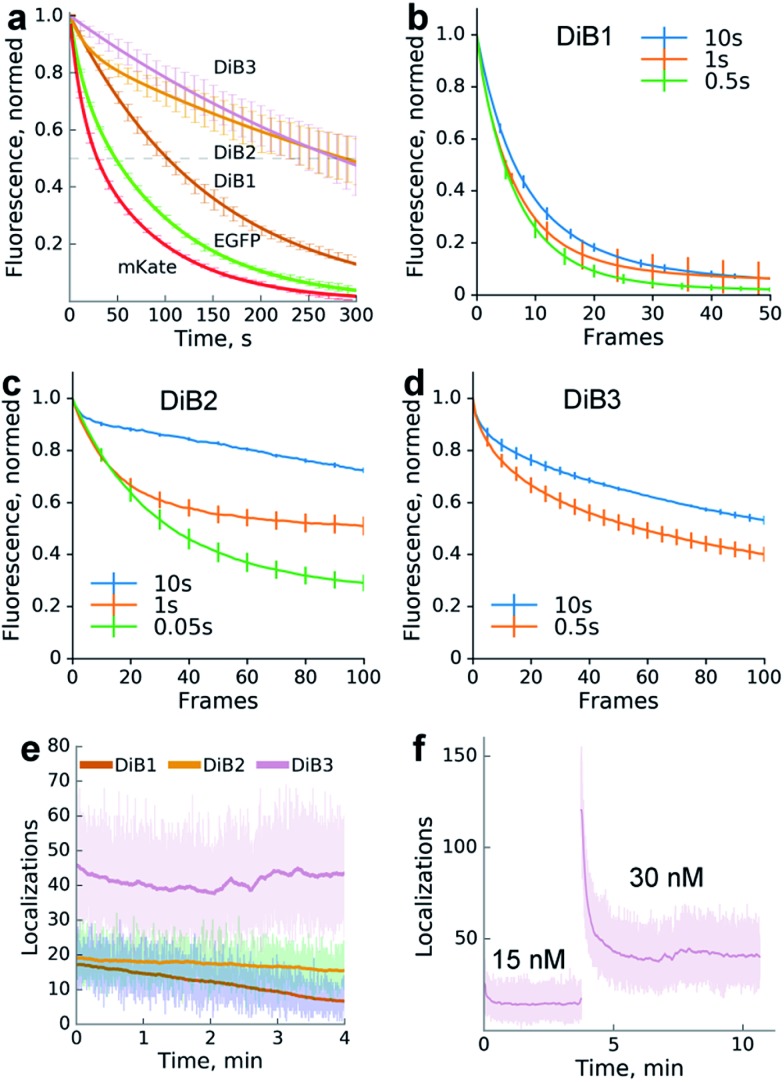
Photostability of the protein-PAINT labeling system. Live-cell performance of the protein-PAINT labeling system. (a) Photobleaching curves of HEK293T expressing H2B fused with Blc mutants and stained with chromophore **M739** in a confocal setup. Curves for H2B-EGFP and H2B-mKate under the same imaging conditions are provided for comparison. mKate and **DiB3**: a 55.4 μm^2^ region was scanned with a 20 μW 543 nm laser. EGFP, **DiB2** and **DiB1**: a 3542 μm^2^ region was scanned with a 100 μW 488 nm laser. Lines – two-term exponential fitting; error bars – s.d. (b–d) The impact of the imaging regime on **DiB**s photostability. Plots show the photobleaching curves obtained by collecting widefield images with different time gaps between ‘bleaching’ frames ((b and d) 1 s and (c) 0.1 s bursts of ∼60 W cm^–2^ light) for live HEK293T cells expressing H2B fused with **DiB1**, **DiB2**, and **DiB3** in the presence of 0.5, 5, and 10 μM **M739**, respectively. (e and f) Photostability in the single-molecule imaging (TIRF) setup. The graph shows the number of localizations per frame (an ∼50 μm^2^ region of the frame is illuminated with 4.5 W cm^–2^ 488 nm or 120 W cm^–2^ 561 nm laser light in TIRF mode). The laser illumination occurred without intermittence, and the frames were taken with 16 ms exposure. (f) Prolonged single molecule imaging with **DiB3** in the presence of 15 and 30 nM **M739**. Note that the addition of the chromophore solution to the cell medium immediately results in an increased number of localization events.

We compared the photostability of the fluorescence signal of **DiB**-labeled (1 μM **M739**) histone H2B in living cells with the photostability of spectrally similar fluorescent proteins. We found that the protein–fluorogen pairs with higher *K*_d_ values (**DiB2** and **DiB3**) exhibited an order of magnitude higher photostability than the corresponding fluorescent proteins (Fig. 2a), in agreement with the original idea of a dye exchange-based photostability increase.^28^ Also, the washout time for **DiB1** observed in labeling-washout cycles in live cells was notably higher than for **DiB2** (Fig. 1c). By imaging the washout of fixed detergent-treated cells with a higher temporal resolution we estimated the apparent *k*_off_ values of the **DiB**s to be 0.01 s^–1^ for **DiB1**, 0.1 s^–1^ for **DiB2**, and 0.3 s^–1^ for **DiB3**. We also observed the positive effect of the delay between the frames on the overall photostability (Fig. 2b–d), even at fluorogen concentrations far above the *K*_d_ (Fig. 2b). Prolonged time-series with no sample illumination in between the frames is a typical live-cell microscopy setup, for which labeling with **DiB**s may be particularly useful.

To further test the utility of the **DIB**s we analysed their performance in single-molecule localization microscopy. We expected that application of the dye at a low concentration far below the *K*_d_ value would result in the stochastic formation of dye–protein complexes which would be detectable as sparse single-molecule fluorescence bursts, similarly to the FAP,^28^ PAINT^13^ or DNA-PAINT^16^ methods.

We therefore performed TIRF imaging of **DiB**-labeled cytoskeletal proteins (cytokeratin, α-actinin and vimentin) in live HeLa Kyoto cells with an EM-CCD camera, allowing for the detection of single molecule emitters (Fig. 2f).

**Fig. 3:**
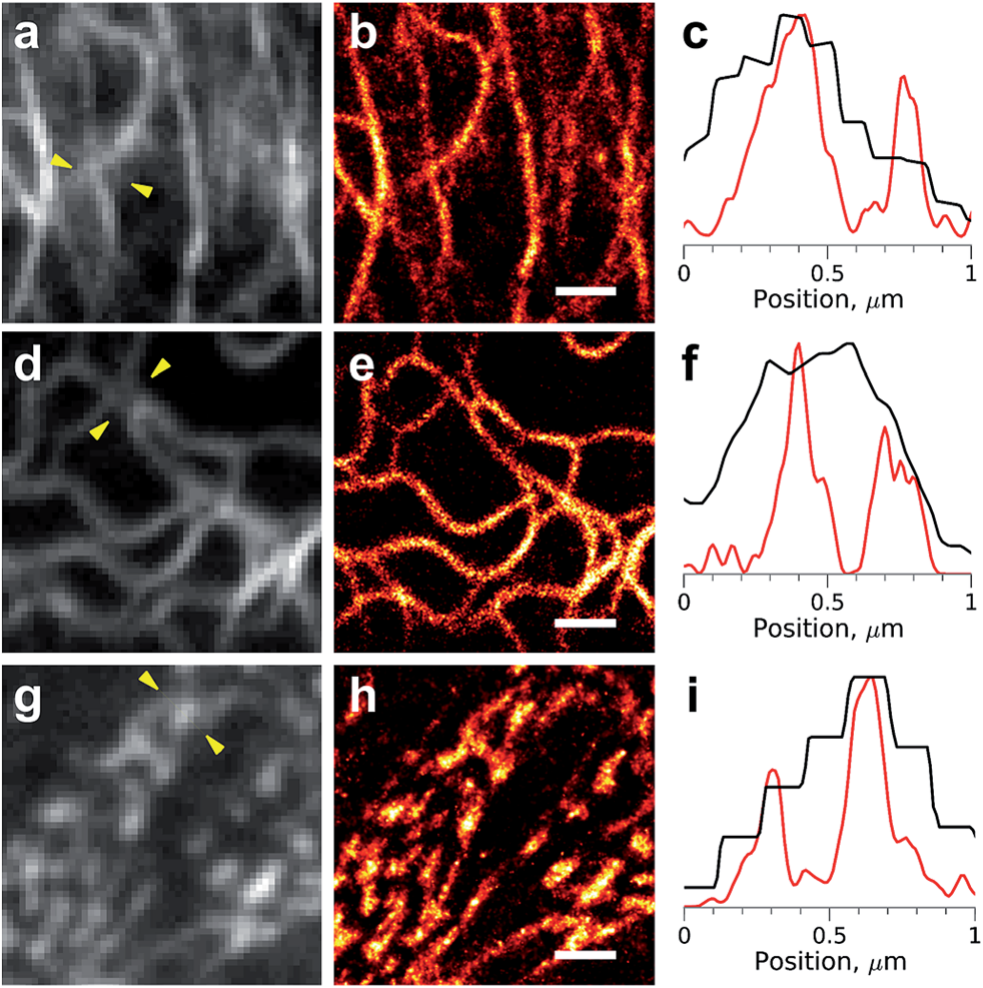
Super-resolution imaging with the protein-PAINT labeling system under a moderate illumination power. Live HeLa Kyoto cells were transiently transfected with cytokeratin-**DiB1** (a–c), vimentin-**DiB2** (d–f) or α-actinin-**DiB3** (g–i). Widefield images (a, d and g) and super-resolution reconstructions (b, e and h) from 5000 frames are shown; scale bars are 1 μm. (c, f and i) Normalized intensity profiles between the arrowheads shown on the widefield images (a, d and g); black curves – widefield and red curves – super-resolution. The fluorescence was excited with 488 nm (4.5 W cm^–2^, (a–f)) or 561 nm (120 W cm^–2^, (g–i)) laser lines.

Addition of the compound **M739** at nanomolar concentrations led to the immediate appearance of sparsely distributed individual fluorescence spots (ESI Movies and Dataset†). The number of detected fluorescence bursts per frame increased with the increase in dye concentration (Fig. 2f). Remarkably, a dynamic equilibrium between dye entry into the cell, **DiB** complex formation, dissociation and bleaching resulted in a constant number of events per frame for **DiB2** and **DiB3** at the tested concentrations of the dye. In contrast, the number of **DiB1** emitters declined rapidly in similar conditions (Fig. 2e), in accordance with the photostability data (Fig. 2a). The observed temporal stability of **DiB2** and **DiB3** labeling at the single-molecule level is in contrast with the previously reported time-traces of fluorogen activation by FAP,^28^ in which ever-increasing concentrations of fresh fluorogen were required for the same effect. Taken together, the **DiB**s' photostability data (at the single-molecule detection level, in a widefield and confocal mode) indicate the possible link between the residence time (determined by *k*_off_) of the fluorogen within the complex and the irreversible photobleaching. Indeed, photobleaching of GFP-like proteins occurs through multistep photochemical reactions of the chromophore, amino acids of the protein host and external compounds.^30^ With a number of possible long-lived and optically active fluorogen intermediates, the release of at least some of them into the solution before permanent damage of the protein host would improve photostability. The link between the photostability and the *k*_off_ was also reported for some FAP variants.^31^ While all of the **DiB**s could be used for single molecule localization microscopy (Fig. 3 and S6†), only the low-affinity pairs (**DiB2** and **DiB3**) exhibited the full advantages of the dynamic dye interchange. Thus, the protein-PAINT labeling method with **DiB2** and **DiB3** allows for the prolonged super-resolution imaging of live cells with the density of single-molecule fluorescence bursts controlled by the concentration of the dye in the imaging solution.

Importantly, a significant increase in image resolution (Fig. 3, right column) could be achieved with illumination intensities far below damaging levels,^29^ typical for most of the single-molecule localization microscopy techniques. However, higher photon numbers could be obtained, if needed, with an increase of the laser power (Fig. S7†). The high photostability of the **DiB**s is also beneficial for other light-intensive super-resolution modalities, such as stimulated emission depletion (STED). We observed a dramatic increase in the photostability (in comparison with a fluorescent protein) of **DiB1** and **DiB2** in STED mode on the same scale (Fig. S8†) as in the confocal mode (Fig. 2a).

## Conclusions

To conclude, we engineered novel fluorogen–protein pairs exhibiting high photostabilities which were well-suited for single-molecule localization super-resolution microscopy. The fluorogenic GFP-like chromophores tested in this work showed rapid cell penetration and reversible labeling, allowing for simple on-demand tuning of the labeling density in living cells. Fluorogenic dyes greatly simplify cell staining, allowing for more sophisticated experimental design: in principle, various fluorogen-based labeling systems could be combined in the same experiment, with temporal separation in a single fluorescence channel, similarly to multiplexed DNA-PAINT,^17^,^18^ but in live cells. The Blc lipocalin, naturally evolved to bind various small molecules, proved to be a promising scaffold for the development of fluorogen-based labeling systems.

## Conflicts of interest

M. S. B., I. V. Y. and K. A. L. are the inventors the patent application covering the fluorogenic dyes used in this paper (WO2014031021 A1 and US20150177254 A1).

## Supplementary Material

Supplementary movie

Supplementary movie

Supplementary movie

Supplementary information
