# Supplementary material for: Protein labeling for live cell fluorescence microscopy with a highly photostable renewable signal
Source: Chem Sci. 2017 Aug 3;8(10):7138–42. doi: 10.1039/c7sc01628j (PMC5676496; doi:10.1039/c7sc01628j)
Supplement: Supplementary file 4 [file SC-008-C7SC01628J-s004.pdf]

Electronic Supplementary Material (ESI) for Chemical Science.
This journal is © The Royal Society of Chemistry 2017

Supplementary information

Protein labeling for live cell fluorescence microscopy with a highly photostable renewable signal

Nina G. Bozhanova[a,b], Mikhail S. Baranov[a], Natalia V. Klementieva[b], Karen S. Sarkisyan[a,c], Alexey S. Gavrikov[a], Ilia V. Yampolsky[a,d], Elena V. Zagaynova[b], Sergey A. Lukyanov[a,b,d], Konstantin A. Lukyanov[a,b] and Alexander S. Mishin*[a,b]

[a] Shemyakin-Ovchinnikov Institute of Bioorganic Chemistry, Moscow, Russia
[b] Nizhny Novgorod State Medical Academy, Nizhny Novgorod, Russia
[c] Centre for Genomic Regulation (CRG), The Barcelona Institute for Science and Technology, Dr. Aiguader 88, 08003 Barcelona, Spain.
[d] Pirogov Russian National Research Medical University, Moscow, Russia
*mishin@ibch.ru

Supplementary data (online)

Movie S1 (single-molecule bursts of vimentin-DiB1)
Movie S2 (single-molecule bursts of vimentin-DiB2)
Movie S3 (single-molecule bursts of vimentin-DiB3)
Datasets: https://doi.org/10.6084/m9.figshare.4867139.v2

- 3000-frames extracts from the raw data for Figure S6 and Movies S1-3: dib1-stack.tif, dib2-stack.tif, dib3-stack.tif
- 18000-frames extracts from the raw data for Figure S7: dib1_5kW_cm2.tif, dib2_5kW_cm2.tif

Supplementary figures and tables

Figure S1 S2
Figure S2 S3
Figure S3 S4
Figure S4 S5
Figure S5 S6
Figure S6 S7
Figure S7 S8
Figure S8 S9
Table S1 S10

Methods

Synthesis of the chromophore library S11
Molecular docking S15
Molecular cloning
Protein expression and purification
Cell culture and transient transfection S16
Fluorescence microscopy
Chromophore titration S17
Analysis of fluorescence titration data S18
Quantum yield measurements S19

Supplementary references

Supplementary references S20

a

```
          1         11        21        31        41        51       60
     Blc  MRLLPLVAAA TAAFLVVACS SPTPPRGVTV VNNFDAKRYL GTWYEIARFD HRFERGLEKV
    1QWD  MSYYHHHHHH LESTSLYKKS SS→
  Blc-WT  MGGSHHHHHH LESTSLYKKS SS→

         61        71        81        91       101       111      120
     Blc  TATYSLRDDG GLNVINKGYN PDRGMWQQSE GKAYFTGAPT RAALKVSFFG PFYGGYNVIA

        121       131       141       151       161       171   177
     Blc  LDREYRHALV CGPDRDYLWI LSRTPTISDE VKQEMLAVAT REGFDVSKFI WVQQPGS*
```

b

In silico screening ID	Amino acid substitutions	
(**DIB1**) 16912	A36C	L141N
(**DIB2**) 16912a	A36C	-
16912b	-	L141N
(**DIB3**) 78776	V74F	L141Q
78776a	V74F	-
78776b	-	L141Q
78762	V74F	L141G
92308	A93C	L141G
67598	A62S	L141G
76775	V74F	V106F
76778	V74F	V106M
103818	V74F	L104H
76737	V74M	V106W
67501	Y64V	V106W
67499	Y64V	V106F
84378a	-	V106Y
84378b	Q87Y	V106Y
74800	V74Y	N76F
81776	N76T	Y116P

Figure S1. Blc mutants. (**a**) alignment of Blc, crystallised fragment of Blc (PDB ID: 1QWD), and the construct used in this work referred to as ‘Blc-WT’. Only N-terminal parts differ (mismatches are highlighted in blue); sequences are identical after the arrow symbol. Amino acid positions facing the pocket are shaded. (**b**) Amino acid substitutions of the Blc-WT mutants tested *in vitro*.

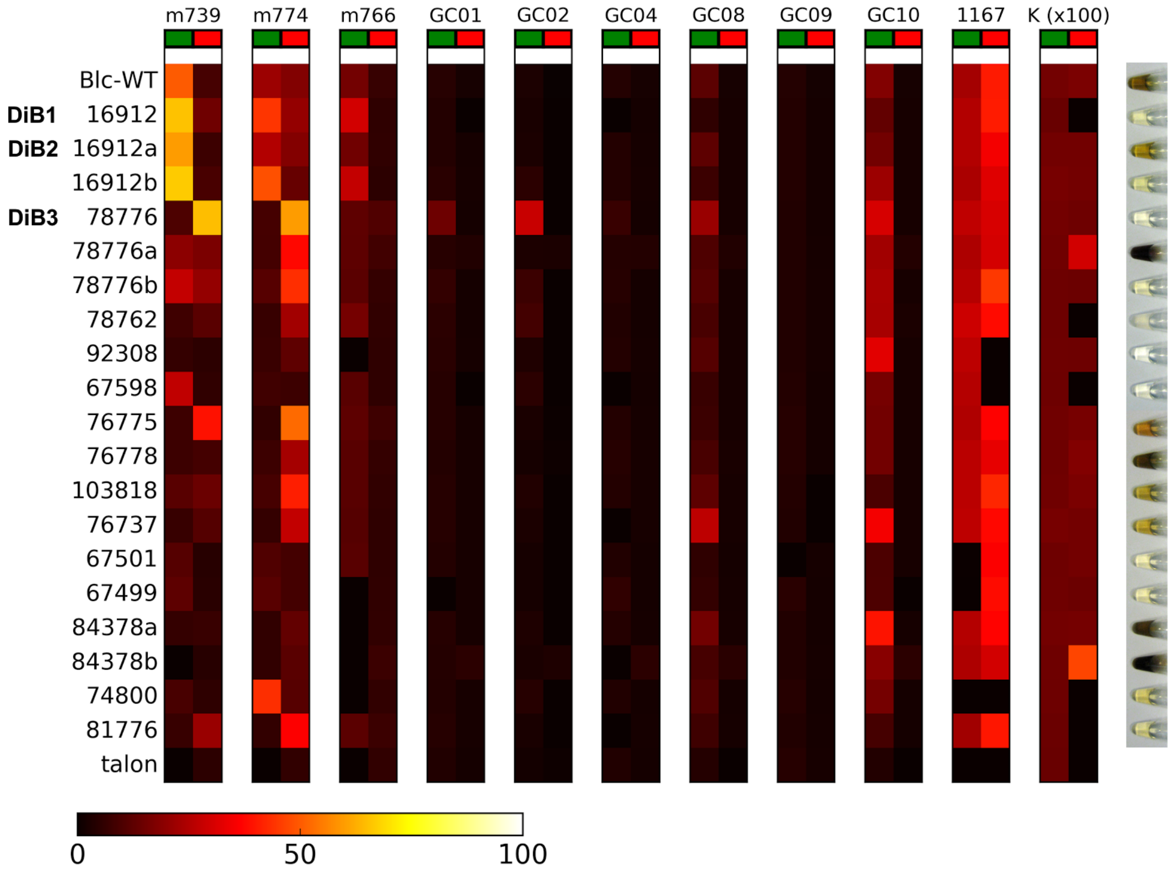

Figure S2. *In vitro* validation of protein-chromophore pairs. Microscopy-based fluorescence assay. Each datapoint corresponds to an experiment with one chromophore-protein pair. Median intensity of 50-200 Talon beads with immobilized protein is shown in pseudocolor. Top row - list of chromophores. Proteins are listed on the left side of the figure. "Talon" in the protein list stands for empty Talon beads. "K" in the list of chromophore stands for PBS buffer without chromophore (exposure is 100x times longer in this case). Only chromophores that showed detectable fluorescence signal are shown. Each vertical bar consists of two columns, corresponding to GFP (marked with green) and TxRed (marked with red) filter sets. The last column is the photograph of protein solutions (at 2 mg/ml) co-purified with unknown bacterial colored compounds.

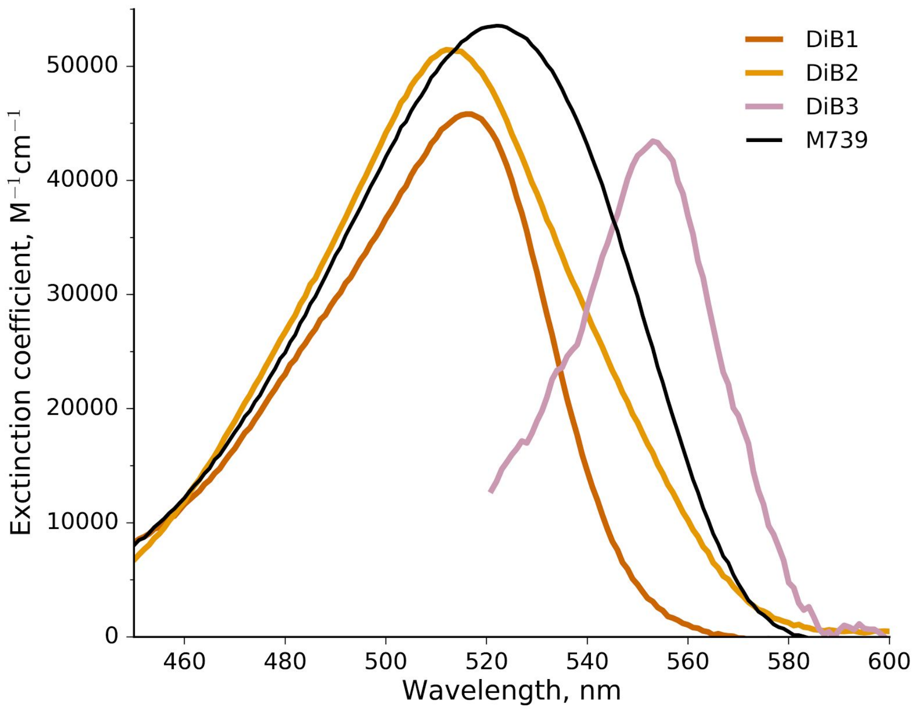

Figure S3. Absorbance of the DiB complexes. Curves for the **DiB1, DiB2, DiB3** show absorbance spectra of the complexes in an excess of the protein, allowing for >90% complex saturation.

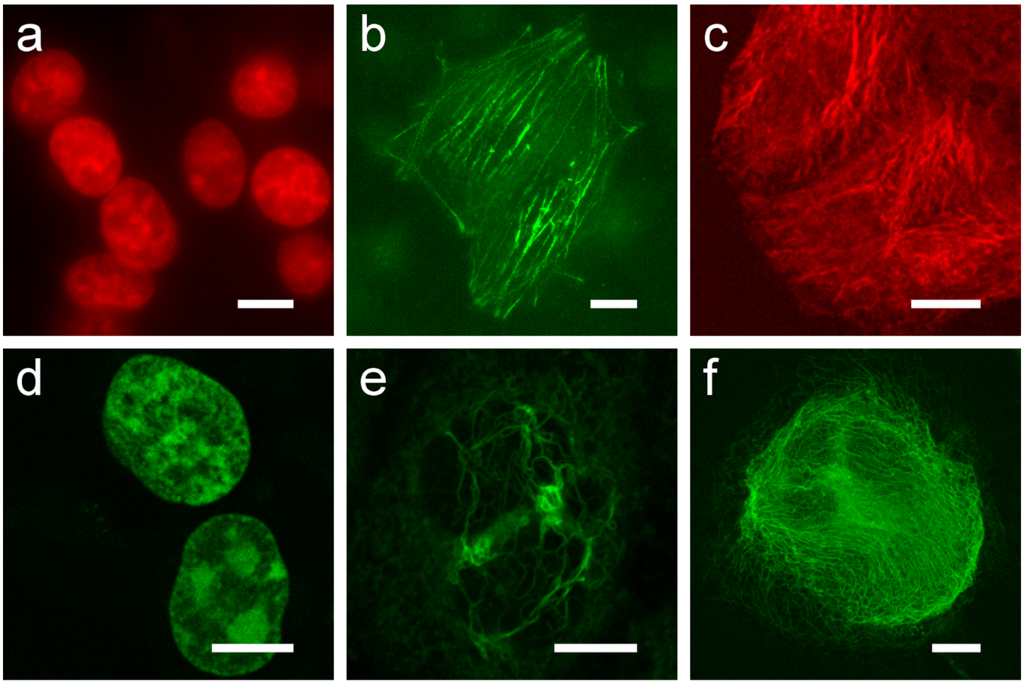

Figure S4. **DiB**s as a protein labelling tag. Transient transfection of HEK293T (**a,d**), NIH-3T3 (**b**), or HeLa Kyoto (**c,e,f**) cell lines with (**a**) H2B-**DiB3** (widefield, TxRed filter set); (**b**) α-actinin-**DiB1** (widefield, GFP filter set); (**c**) α-actinin-**DiB3** (confocal mode, excitation: 543 nm, emission: 560-600 nm); (**d**) H2B-**DiB1** (confocal microscopy, excitation: 488 nm, emission: 520-560 nm); (**e**) vimentin-**DiB2** (confocal microscopy, excitation: 488 nm, emission: 520-560 nm); (**f**) cytokeratin-**DiB1** (confocal microscopy, excitation: 488 nm, emission: 520-560 nm). Scale bars - 10 μm.

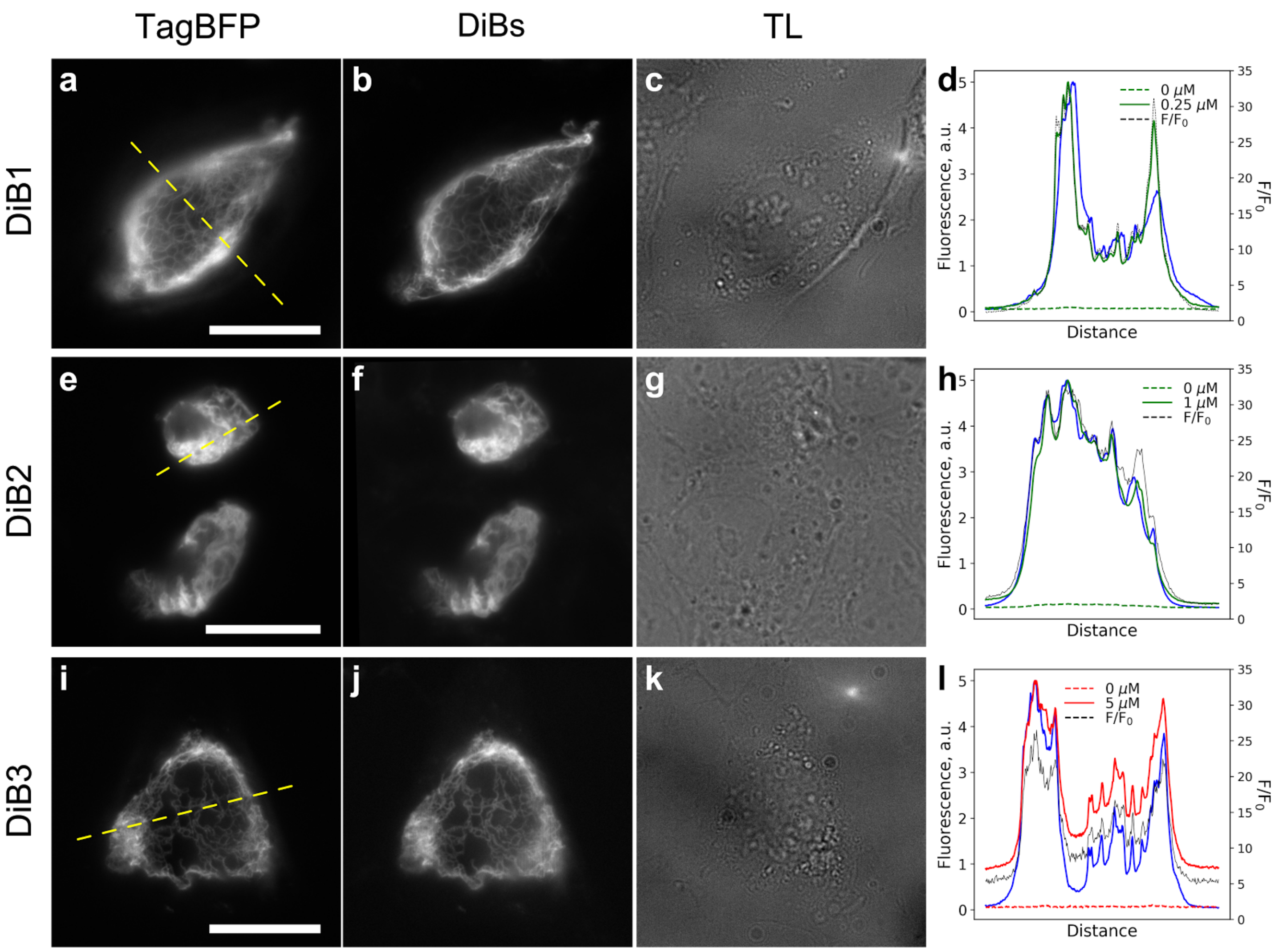

Figure S5. Fluorescence increase and background estimation in live cells. U2OS cells were imaged 16h after transient transfection with vimentin-TagBFP-Blc plasmids (**DiB1**, **DiB2**, **DiB3**). Images were taken with BLUE (**a,e,i**), GFP (**b,f**), and TxRed (**j**) filter sets (Leica) at 200 ms exposure with 405 nm, 465 nm, and 565 nm LED illumination (CoolLED); HC PL APO 63x/1.30 Glycerol-immersion objective and Andor Zyla 5.1 camera were used; images before and 1 min after addition of the **M739** fluorogen (**b**: 250 nM, **f:** 1 μM, **j**: 5 μM) were analyzed; (**c,g,k**) Transmitted light (TL) images of the same fields of view (10ms exposure); (**d,h,l**) Line profiles. Intensities along the dashed yellow lines on **a,e,i** in BFP before chromophore addition (blue line) and GFP/TxRed channels before (colored dashed line) and after (colored solid line) chromophore addition are plotted (left Y axis, rescaled to aid the visualization). Dashed grey line shows the quotient of the fluorescence after and before addition of the fluorogen (right Y axis). Scale bars 20 μm.

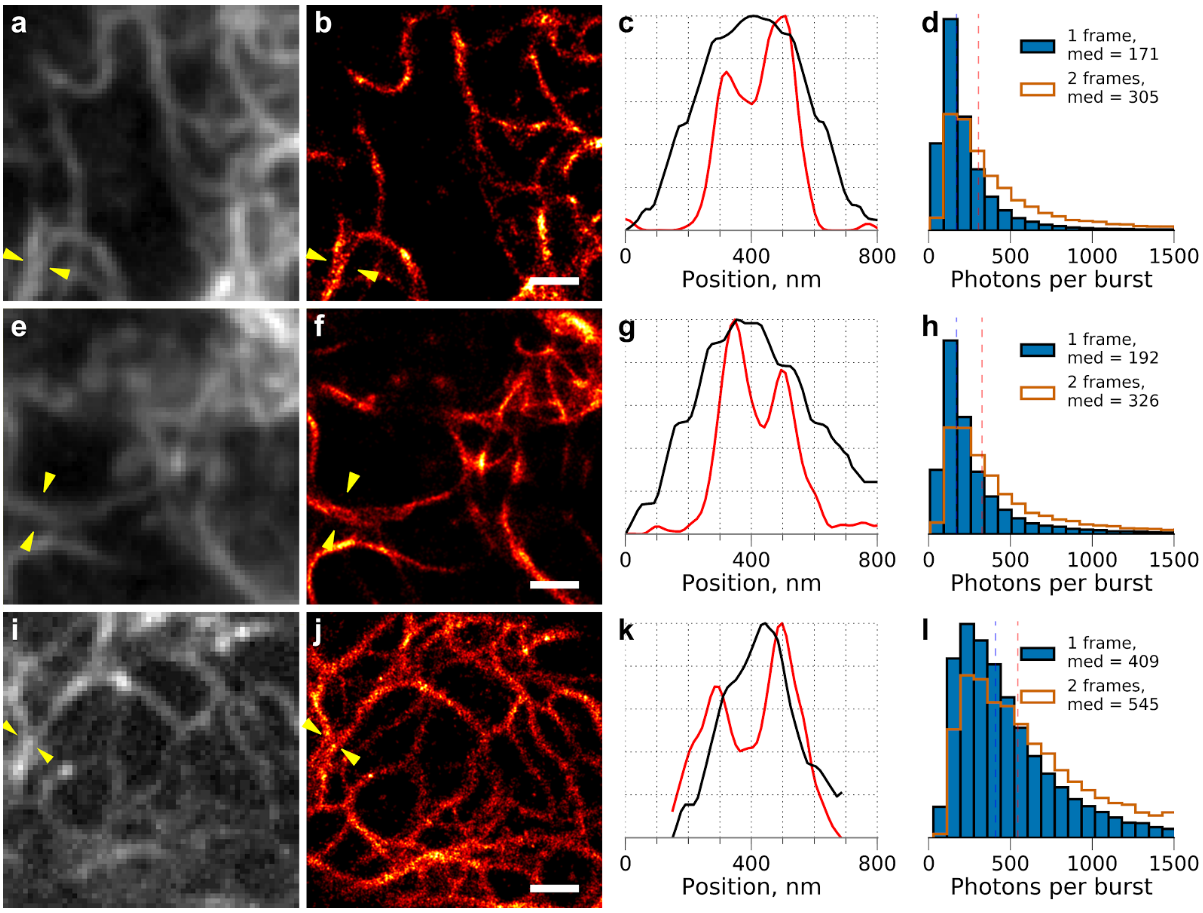

Figure S6. Protein-PAINT Super-resolution microscopy of vimentin tagged with **DiB1** (**a-d**), **DiB2** (**e-h**) or **DiB3** (**i-l**) in live HeLa Kyoto cells. Widefield images (**a,e,i**) and super-resolution (**b,f,j**) reconstructions from 3000-7000 frames; scale bars 1 µm. (**c,g,k**) Normalized intensity profiles between arrowheads shown on the images; black curves - widefield, red curves - super-resolution. (**d,h,l**) Normalized histograms of detected photons per localization per one or two frames. Median values of calculated[1] localization uncertainties are 45, 42, and 35 nm for **DiB1**, **DiB2**, and **DiB3** datasets (available online), as reported by ThunderSTORM[2] software.

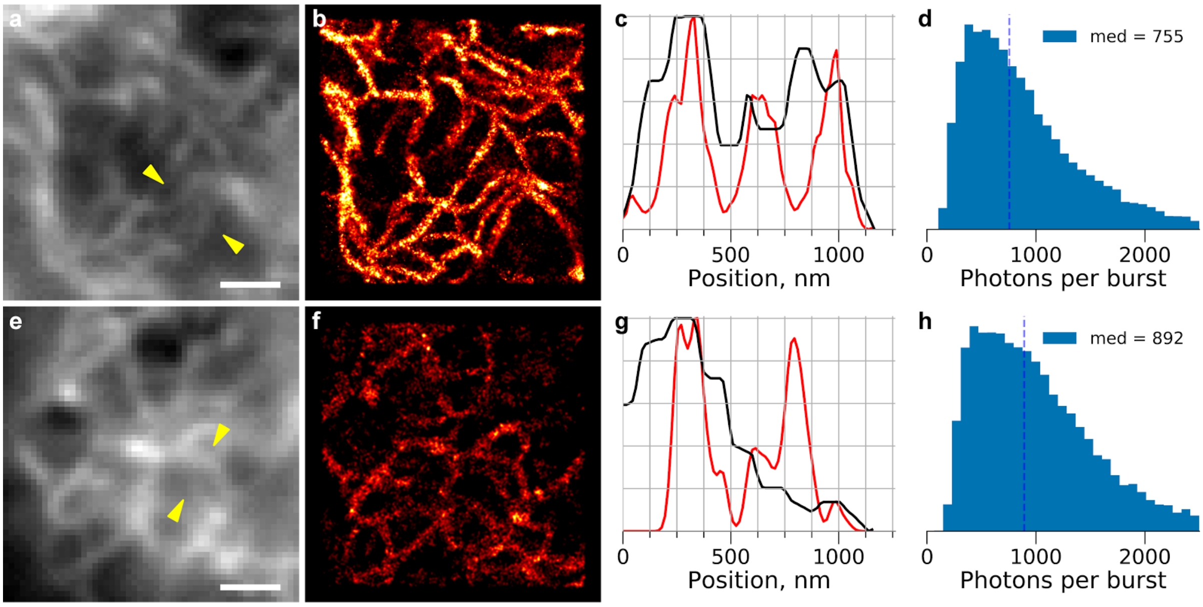

Figure S7. Photon content in high power illumination regime. Vimentin tagged with **DiB1** (**a-d**) and **DiB2** (**e-h**) in live HeLa cells was imaged with ~5 kW/cm^2 of 488 laser on Leica GSDIM microscope. The laser was adjusted to 'epi' mode (straight out of the objective). Cells were incubated with 10 nM **M739** to allow for sparse labelling. Widefield (**a,e**) and super-resolution (**b,f**) images; scale bars 1 μm. (**c,g**) Normalized intensity profiles between arrowheads shown on the images; black curves - widefield, red curves - super-resolution. (**d,h)** Normalized histograms of detected photons per localization per frame (10 ms exposure). Median photon content is indicated. Median values of calculated localization uncertainties are 16 and 18 nm for DiB1 and DiB2 datasets (raw data available online), respectively, as reported by ThunderSTORM[2] software.

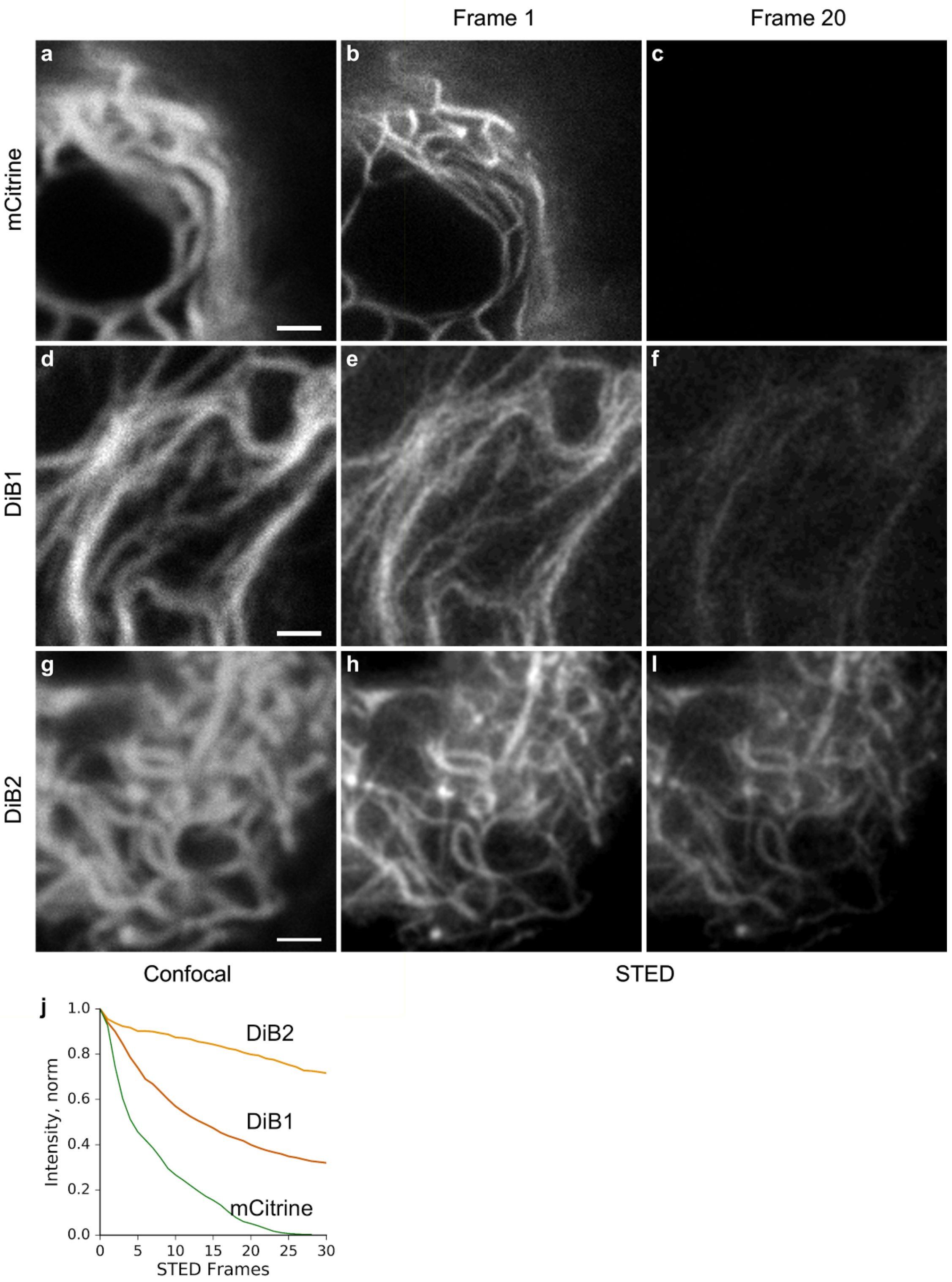

Figure S8. STED super-resolution microscopy with DiBs. Side-by-side comparison of STED of vimentin tagged with fluorescent protein mCitrine (**a-c**) **DiB1** (**d-f**), and **DiB2** (**g-i**) in live HeLa cells. Images were taken first in confocal (a,d,g), then in STED mode (b,c,e,f,h,i) using similar imaging settings: excitation with 40% of 514 nm from White Light Laser operating at 70% output power, depletion with 40% of 1.44 W 592 nm CW laser. (**j**) Normalized mean intensity profiles for STED time series depicted above. The final concentration of 200 nM of **M739** fluorogen was used for **DiB1** and **DiB2** imaging. Scale bars 1 μm.

Table S1. Spectral properties of protein-chromophore complexes.

Chr:Protein (complex name)	λ_{Ex} nm	λ_{Em} nm	FQY, %	K_d, μM
M739 : Blc16912 (**DIB1**)	513	542	32	0.11
M774 : Blc16912	515	545	20	0.46
M766 : Blc16912	n.d.	542	8	0.88
M739 : Blc16912a (**DIB2**)	510	539	32	4.0
M774 : Blc16912a	513	549	11	11.8
M739 : Blc78776 (**DIB3**)	546	565	15	9.05
M774 : Blc78776	552	568	12	4.95
M766 : Blc78776	n.d.	559	15	34.7
M739	520	563	3.5	n.a.
M774	522	568	2.2	n.a.
M766	511	564	5.0	n.a.

FQY - fluorescence quantum yield.
n.d. - not determined.
n.a. - not applicable.

Methods

Synthesis of the chromophore library

General

Commercially available reagents were used without additional purification. For column chromatography E. Merck Kieselgel 60 was used. Thin layer chromatography (TLC) was performed on silica gel 60 F_{254} glass-backed plates (MERCK). Visualization was effected by UV light (254 or 312 nm) and staining with $KMnO_4$.

NMR spectra were recorded on a 700 MHz Bruker Avance III NMR at 293 K. Chemical shifts are reported relative to residue peaks of $CDCl_3$ (7.27 ppm for ^{1}H and 77.0 ppm for ^{13}C) or DMSO-d_6 (2.51 ppm for ^{1}H and 39.5 ppm for ^{13}C). Melting points were measured on a SMP 30 apparatus. High-resolution mass spectra (HRMS) spectra were recorded on an Agilent 6224 TOF LC/MS System (Agilent Technologies, Santa Clara, CA, USA) equipped with a dual-nebulizer ESI source and on a Bruker micrOTOF II instrument.

Synthesis of the compounds GA and GC

Compounds GA and GC were synthesized using typical procedures by general methods I-III (Scheme). The synthetic procedures for all novel compounds are presented directly.

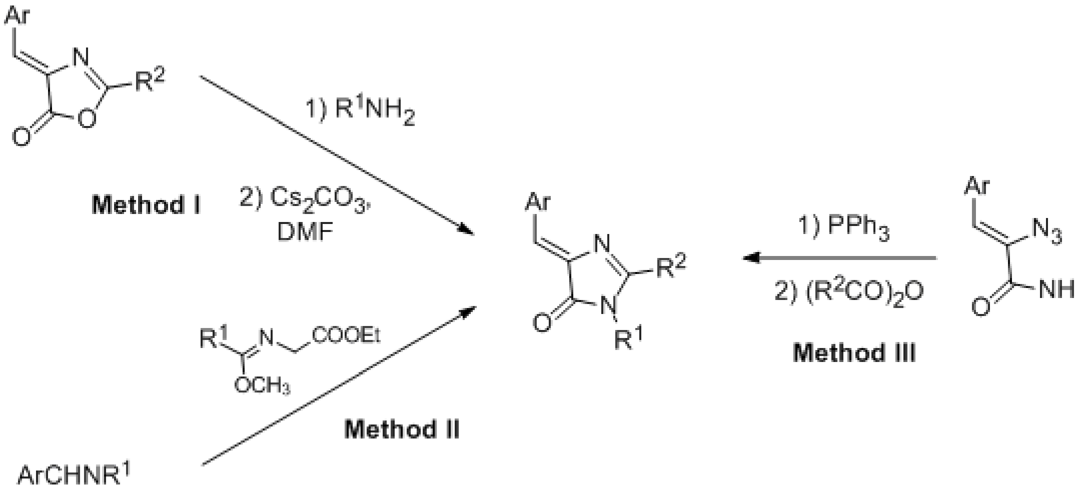

Scheme S1. General methods used in synthesis

General method I

Corresponding aldehyde (50 mmol), amidoacetic acid (60 mmol) and sodium acetate (100 mmol) were dissolved in corresponding anhydride (50 mL). The mixture was stirred at 110°C for 5 hours. The mixture was dissolved by EtOAc (400 mL), washed with water (2x150 mL) and brine (2x150 mL) and dried over Na_2SO_4. The solvent was evaporated and the product was purified by flash chromatography (Hexane-EtOAc). The residue was dissolved in ethanol (100 mL) and the methylamine solution (40% aq) was added. The mixture was stirred for 4 hours and the solvent was evaporated. The dimethylformamide (50 mL) and

Cs_2CO_3 7.0 g (21 mmol) were added and the mixture was refluxed for 10 minutes. The solvent was evaporated and the mixture was dissolved by EtOAc (300 mL), washed with water (2x100 mL) and brine (2x100 mL) and dried over Na_2SO_4. The solvent was evaporated and the product was purified by column chromatography ($CHCl_3$-EtOH).

General method II

Corresponding aldehyde (20 mmol) was mixed with 5 mL of 40% aqueous methylamine solution, anhydrous sodium sulfate (20 g) and chloroform (100 mL). The mixture was stirred for 48 hours at room temperature, filtered and dried over the additional Na_2SO_4. The solvent was evaporated and ethyl ((1-methoxy)amino)acetate (3.5 g, 22 mmol) and ethanol (10 mL) were added. The mixture was stirred for 24 hours at room temperature, solvents were removed in vacuum and the product was purified by column chromatography ($CHCl_3$-EtOH).

General method III

A solution of azido-cynnamate (10.0 mmol) and triphenylphosphine (2.9 g, 11.1 mmol) in dry toluene (50 mL) was heated to 65°C under argon. Yellow precipitate formed and effervescence appeared. After 30 minutes reaction mixture was cooled to room temperature and anhydride (20 mmol) and DIPEA (10 mmol) was added. The mixture was heated to 65°C again and stirred. The reaction mixture was cooled, and dissolved by chloroform (100 mL), washed with $NaHCO_3$ solution (5%, 100mL), water (2 x 50 mL), brine (2 x 50 mL) and dried over Na_2SO_4. The solvent was evaporated and the product was purified by column chromatography ($CHCl_3$:EtOH).

Table S2. The structure of compounds **GA**.

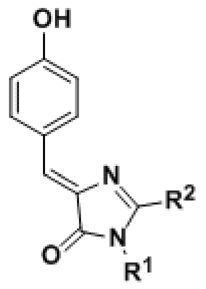

GA

Compound number	R^2	R^1	Synthetic method	reference
**GA01**	H	CH_3	III[a]	[3]
**GA02**	CH_3	H	II	[3]
**GA03**	CH_3	CH_3	II	[3]
**GA04**	CH_3	Et	III[a]	[3]

**GA05**	CH_3	Pr	III[a]	[3]
**GA06**	CH_3	iPr	III[a]	[3]
**GA07**	CH_3	tBu	III[a]	[3]
**GA08**	CH_3	Ph	III[a]	[3]
**GA10**	CH_3	CF_3	III[a]	[3]
**GA17**	OCH_3	Et	I	[4]
**GA18**	CH_3	iBu	I	[4]
**GA19**	OCH_3	iBu	I	[4]

a – synthesized by BBr_3 demethylation from corresponding OCH_3 derivatives, see [3]

Table S3. The structure of compounds **GC**.

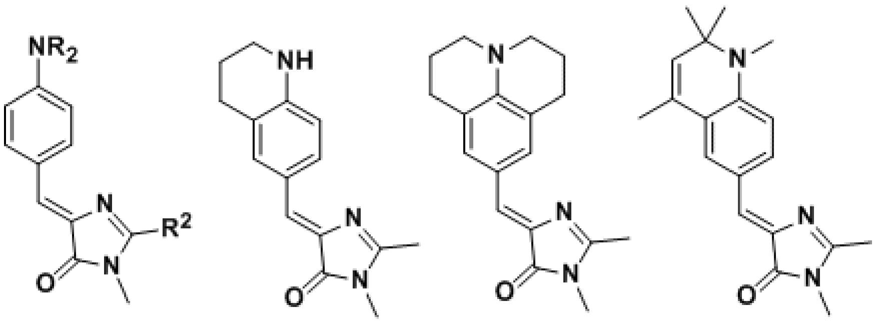

GC GC08 GC09 GC10

Compound number	R^2	R	Synthetic method	reference
**GC01**	CH_3	Et	II	[5]
**GC02**	Et	Et	I	Novel, see below
**GC04**	CH_3	CH_3	II	[5]
**GC05**	CH_3	H	II[a]	[5]
**GC06**	CH_3	n-Bu	II	[5]
**GC08**	CH_3	See above	II	[5]
**GC09**	CH_3		II	[5]
**GC10**	CH_3		II	[5]

a – synthesized from compound **GB08** by reduction, see [5]

(Z)-4-(4-(diethylamino)benzylidene)-2-ethyl-1-methyl-1H-imidazol-5(4H)-one (GC02).

4-(diethylamino)benzaldehyde 8.85 g (50 mmol), 2-propionamidoacetic acid 7.86 g (60 mmol) and sodium acetate 6.6 g (100 mmol) were dissolved in propionic anhydride (50 mL)/ The mixture was stirred at 110°C for 5 hours. The mixture was dissolved by EtOAc (400 mL), washed with water (2x150 mL) and brine (2x150 mL) and dried over Na_2SO_4. The solvent was evaporated and the product was purified by flash chromatography (Hexane- EtOAc 4:1). The residue was dissolved in ethanol (100 mL) and the methylamine solution (40% aq) was added. The mixture was stirred for 4 hours and the solvent was evaporated. The dimethylformamide (50 mL) and Cs_2CO_3 7.0 g (21 mmol) were added and the mixture was refluxed for 10 minutes. The solvent was evaporated and the mixture was dissolved by EtOAc (300 mL), washed with water (2x100 mL) and brine (2x100 mL) and dried over Na_2SO_4. The solvent was evaporated and the product was purified by column chromatography ($CHCl_3$-EtOH 10:1).

Orange solid (5.99 g, 42%); mp = 141-144°C; ^{1}H NMR (DMSO-d_6) δ 8.07 (d, J=8.6 Hz, 2H), 6.85 (s, 1H), 6.72 (d, J=8.6 Hz, 2H), 3.42 (q, J=7.0 Hz, 4H), 3.07 (s, 3H), 2.65 (q, J=7.2 Hz, 2H), 1.26 (t, J=7.2 Hz, 3H), 1.12 (t, J=7.0 Hz, 6H); ^{13}C NMR (DMSO-d_6) δ 9.2 (CH_3), 12.4 (CH_3), 21.2 (CH_2), 25.8 (CH_2), 43.7 (CH_3),111.0 (CH), 120.9, 126.5 (CH), 134.1 (CH), 134.2, 148.7, 163.4, 169.8; HRMS (ESI) m/z: 286.1921 found (calcd for $C_{17}H_{24}N_3O$, [M+H]+ 286.1919).

Synthesis of the conformationally-locked compounds ABDI-BF_2

ABDI-BF_2 compounds were synthesized as reported previously [5] by the direct borylation of the corresponding imidazolones **GC** (Scheme S2). The synthetic procedures for all novel compounds are presented directly.

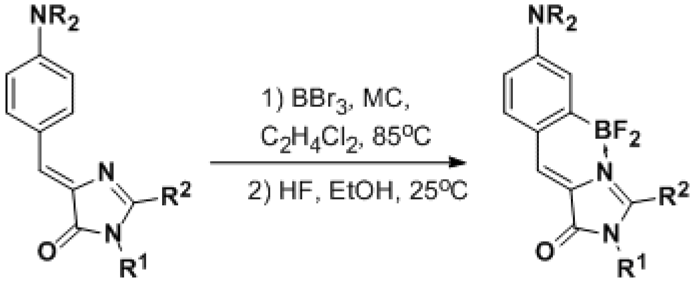

Scheme S2. General procedure of the borylation.

General method of ABDI-BF_2 synthesis

Corresponding imidazol-5(4H)-one (**GC**) (10 mmol) was dissolved in dry CH_2Cl_2 (100 mL), molecular sieves 4Å (10 g) and 3Å (10 g) were added, followed by a solution of borone tribromide in CH_2Cl_2 (1M, 50 mL, 50.0 mmol) and the reaction mixture was refluxed for 2-8 hours in inert atmosphere. The mixture was cooled and filtered; molecular sieves were washed two times by ethanol (50 mL) and CH_2Cl_2 (300 mL). The solution was mixed with aqueous HF (20%, 10 mL) and stirred for 30 minutes. The mixture was dissolved by EtOAc (250 mL), washed with potassium carbonate solution (5%, 2x50mL), water (2x50 mL) and

brine (2x50 mL) and dried over Na_2SO_4. The solvent was evaporated and the product was purified by column chromatography ($CHCl_3$-EtOH).

Table S4. The structure of compounds **ABDI-BF_2**.

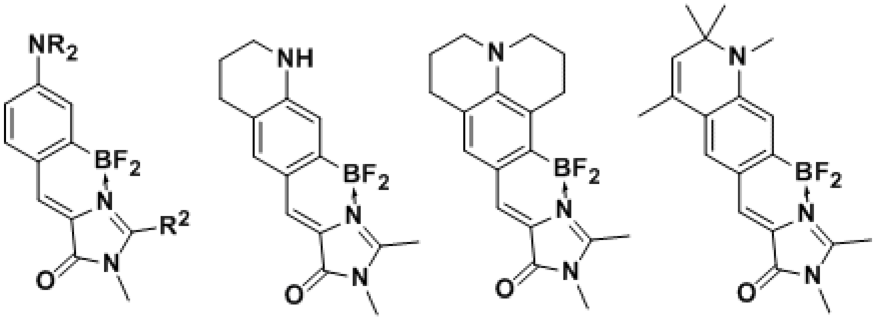

ABDI-BF_2 M1167 M1035 M1169

Compound number	R^2	R	reference
**M739**	CH_3	Et	[5]
**M774**	Et	Et	Novel, see below
**M766**	CH_3	CH_3	[5]
**M816**	CH_3	H	[5]
**M1078**	CH_3	n-Bu	[5]
**M1035**	CH_3	See above	[5]
**M1169**	CH_3		[5]
**M1167**	CH_3		[5]

(Z)-4-(4-(diethylamino)-2-(difluoroboryl)benzylidene)-2-ethyl-1-methyl-1H-imidazol-5(4H)-one (M774).

(Z)-4-(4-(diethylamino)benzylidene)-2-ethyl-1-methyl-1H-imidazol-5(4H)-one (**GC02**) 1.43 g (5.0 mmol) was dissolved in dry CH_2Cl_2 (50 mL), molecular sieves 4Å (4 g) and 3Å (4g) were added, followed by a solution of boron tribromide in CH_2Cl_2 (1M, 20 mL, 20.0 mmol) and the reaction mixture was refluxed for 6 hours in inert atmosphere. The mixture was cooled and filtered; molecular sieves were washed two times by ethanol (20 mL) and CH_2Cl_2 (100 mL). The solution was mixed with aqueous HF (20%, 5 mL) and stirred for 30 minutes. The mixture was dissolved by EtOAc (100 mL), washed with potassium carbonate solution (5%, 2x50mL), water (2x50 mL) and brine (2x50 mL) and dried over Na_2SO_4. The solvent was evaporated and the product was purified by column chromatography ($CHCl_3$-EtOH 10:1).

Red solid (1.28 g, 77%); mp = 183-185°C; ^1H NMR (DMSO-d_6) δ 7.49 (s, 1H), 7.43 (d, J=8.8 Hz, 1H), 6.86 (d, J=2.2 Hz, 1H), 6.66 (dd, J_1=8.8 Hz, J_2=2.2 Hz, 1H), 3.47 (q, J=7.0

Hz, 4H), 3.26 (s, 3H), 3.05 (q, J=7.7 Hz, 2H), 1.29 (t, J=7.7 Hz, 3H), 1.15 (t, J=7.0 Hz, 6H); ^{13}C NMR (DMSO-d_6) δ 10.6 (CH_3), 12.5 (CH_3), 19.4 (CH_2), 26.2 (CH_2), 43.9 (CH_3), 110.6 (CH), 113.8 (CH), 121.1, 130.4 (CH), 134.7 (CH), 150.5, 162.3, 164.2; HRMS (ESI) m/z: 334.1884 found (calcd for $C_{17}H_{23}BF_2N_3O$, [M+H]+ 334.1902).

Molecular docking

For preliminary molecular docking we followed previously published protocol[6]. Briefly, a library of lipocalin structures with two or one substitutions in amino acids facing potential binding site was generated with MODELLER software[7] guided by published lipocalin structure (PDB ID: 1QWD). Files for the ligand (GFP chromophore) and receptor (lipocalin mutants) were prepared for docking in AutoDockTools[8] version 1.5.6. Bounding box for docking was calculated in PyMol. Docking with rigid protein geometry was performed with AutoDock Vina[9].

Molecular cloning

The full copy of the crystallized fragment of the Blc protein[10] was made in two stages. First, the shortened *blc* gene was amplified by nested polymerase chain reaction (PCR) from *E. coli* strain K12 Rosetta-gami 2(DE3)pLysS (external primer pair: Blc-F: 5'-ATGCCGTGTGGACGGGTATT-3', Blc-R: 5'-CGCCGCCTGGCAGATTCTTA-3'; internal primer pair: Blc-Nhe-F: 5'-GTAC<u>GCTAGC</u>AGCTCCACGCCGCCGCGTGGCGTGAC-3', Blc-Hind-R: 5'-GTAC<u>AAGCTT</u>CTAACTACCAGGCTGCTGTACCCAAATA-3'). The PCR fragment was cut with NheI/HindIII and ligated into NheI/HindIII-digested iRFP-pBAD vector instead of iRFP coding sequence. Then the missing linker sequence (Supplementary Figure 1a) between His-tag and the Blc protein fragment was added by self-assembling cloning [11] (Blc-link-F: 5'-AAGAAATCCAGCTCCACGCCGCCGC-3', Blc-link-F-long: 5'-ACCAGCTTGTACAAGAAATCCAGCTCCACGCCGCCGC-3', Blc-link-R: 5'-TGATTCGAGATGATGATGATGATGATGAGAACCC-3', Blc-link-R-long: 5'-GTACAAGCTGGTTGATTCGAGATGATGATGATGATGATGAGAACCC-3'). Coding sequence of Blc-pBad was checked by sequencing and this vector was used for further work.

All 19 selected mutants differ from Blc in one or two amino acids. They were constructed either by one or by two rounds of self-assembling cloning[11].

The H2B-TagBFP-Blc-mutant, actinin-Blc-mutant, and keratin-Blc-mutant plasmids for expression in mammalian cells were constructed by self-assembling cloning using Blc-mutant-pBAD and H2B-TagBFP (Evrogen), keratin-TagRFP (Evrogen) or actinin-TagRFP (Evrogen) vectors as a template.

The actinin-TagBFP-Blc-mutant and vimentin-TagBFP-Blc-mutant plasmids were made by standard digestion-ligation cloning approach. The TagBFP-Blc-mutant inserts were cut with AgeI/NotI from the H2B-TagBFP-Blc-mutant plasmids and ligated into AgeI/NotI-digested actinin-TagRFP (Evrogen) or vimentin-Citrine vector.

Protein expression and purification

Blc and Blc-mutant proteins were expressed in XJb(DE3) Autolysis (Zymo Research) E. coli strain. One bacterial colony was inoculated into 200 ml of LB broth and grown overnight (~

16 hours) at 37°C. Then 800 µl of 20% L-arabinose aqueous solution were added. After another 24 hours at 37°C cells were harvested and resuspended in 5 ml of PBS buffer (pH 7.4). Suspension was frozen at -70°C and thawed at 37°C three times. DNA was destroyed by short sonication, the lysate was centrifuged to obtain cell-free extract. The protein was purified using TALON metal affinity resin (Clontech). A fraction of beads with immobilized protein were aliquoted into a different tube while the rest was washed with PBS / 0.1M EDTA buffer (pH 7.4) to elute the protein into the solution. Finally, all purified samples were dialysed against PBS (pH 7.4).

Cell culture and transient transfection

We have constructed a number of plasmids containing fusion proteins with selected Blc-mutants (H2B-TagBFP-blc16912, H2B-TagBFP-blc78776, keratin-blc16912, actinin-TagBFP-blc78776, actinin-blc16912, actinin-blc78776, vimentin-TagBFP-blc16912a, vimentin-TagBFP-blc16912, vimentin-TagBFP-blc78776) which were used for expression in human cell lines (HeLa Kyoto, NIH-3T3, and HEK293T) and subsequent confocal and wide-field fluorescence microscopy.

HeLa Kyoto, NIH-3T3 and HEK293T cells were grown in Dulbecco's modification of Eagle's medium (DMEM) (PanEco) containing 50 U/ml penicillin and 50 µg/ml streptomycin (PanEco), 2 mM L-glutamine (PanEco) and 10% fetal bovine serum (HyClone, Thermo Scientific) at 37°C and 5% CO_2. The day before transfection the cells were plated in 35mm Fluorodish cell culture dishes (WPI). For transient transfections the FuGENE 6 transfection reagent (Roche) was used according to protocol. Cells were imaged 24 - 48 hours after transfection. Before imaging the cells were washed two times with Versene solution (PanEco) and imaged in Hank's balanced salt solution (PanEco) or minimum essential medium Eagle (MEM) (Sigma), both supplemented with 20 mM HEPES (PanEco).

Fluorescence microscopy

Primary protein-fluorophore interaction screening was performed with TALON beads. Dialysed Blc-mutant protein containing beads (or free TALON beads as a control) were placed in wells of a 96-well plate with 100 µl of PBS buffer. Then the solutions of fluorophores were added to obtain 10 µM concentration. After 10-min incubation at RT the beads were imaged by using BZ-9000 fluorescence microscope (Keyence) with a 2x objective, OP-79301 SB GFP-BP and OP-79302 SB TexasRed filters, and equal exposure time.

Widefield fluorescence microscopy was performed with tthe BZ-9000 fluorescence microscope (Keyence) with a 40x objective (filter sets OP-79301 SB GFP-BP and OP-79302 SB TexasRed). Confocal imaging presented in the Manuscript (Figure 1) as well as the bleaching experiments (Figure 2b) was performed using an inverted Leica confocal microscope DMIRE2 TCS SP2 (Leica, Wetzlar, Germany) equipped with HCX PL APO lbd.BL 63.0x1.40 OIL objective, excitation by 488 nm laser line (100 µW) , detection at 500-530 nm (DiB1, DiB2), or by 543 laser line (20 µW), detection at 550-600 nm (DiB3).

Single-molecule localization super-resolution imaging of living cells was carried out on a Nikon Eclipse Ti N-STORM microscope (Nikon, Japan) controlled by NIS-Elements

Software and/or the Micro-Manager software[12]. The excitation light from 488 nm (4.5 W/cm2) or 561 nm (120 W/cm2) laser lines was focused on the sample with a 100X oil-immersion objective (Apo TIRF/1.49, Nikon) and PFS (perfect focus system). Optical path included C-NSTORM QUAD filter cube (Nikon) and a 1.5x magnifier lens. The images (a pixel size of 107 nm) were captured with the EM-CCD camera (iXon3 DU-897, Andor, UK) at 10 MHz readout rate, 14-bit, with an EM gain and a pre-amplifier gain set to 200 and 5.1 (12.15 electrons per A/D count) respectively, with an exposure time of 16-50 ms. The TIRF illuminator mirror was adjusted to allow for total internal reflection (objective-based TIRF). A typical series of 5000 frames was taken using ND Acquisition mode. The running median filtering was applied to the time series prior to localization step[13]. Sparse images of individual fluorophores were fitted with the ThunderStorm[2] plugin for FIJI[14]. Localization uncertainties reported here are calculated by ThunderStorm[2] following EM-CCD optimized formula[1].

Single-molecule localization with high-power illumination was carried out on Leica SR GSDIM system, equipped with the EM-CCD camera (Andor iXon 3 897), SuMo stabilized stage, HCX PL APO 100x/1.47 OIL objective with additional 1.6x magnification (96 nm effective pixel size). The sample was illuminated with 488 laser line operating in "epi" mode (straight out of the objective") at the power density of 5 kW/cm^2. The camera exposure time was set to 10 ms. Camera settings: 10 MHz readout rate, 14-bit, pre-amplifier gain 5 (12.15 electrons per A/D count), EM gain 296.

STED microscopy was performed on Leica TCS SP8 STED 3X in a Gated-STED mode with Hybrid detectors and HC PL APO CS2 93x/1.30 glycerine-immersion objective. The 514 nm line of the tunable White Light Laser was used for fluorophore excitation at 40% output power. Fluorescence depletion by stimulated emission was achieved with the 40% output power of 1.44W 592 nm continuous-wave laser.

Cell perfusion experiments (Figure 2a) were performed on the Leica AF6000B imaging system equipped with ZYLA-5.5-CL10 camera (Andor, UK), controlled by the Micro-Manager software[12]. The HBSS solution or HBSS with the M739 (0.5 μM) were pumped into the imaging chamber of the μ-Slide VI 0.4 (IBIDI, Germany) with the Milliliter Syringe Pump Module (Harvard Apparatus, USA) controlled via serial port. The excess of the fluid was removed by the peristaltic pump.

Chromophore titration

To investigate the binding of fluorophores to Blc-mutant proteins, fluorescence titration was performed. The data were obtained using newly purificated and dialysed protein, and newly diluted fluorophore solutions.

Samples of dry fluorophore were diluted in DMSO. These solutions were used for subsequent preparation of the 1000X stock solutions in 96% EtOH. The concentration of the fluorophores in the stock solutions was measured using Cary 100 UV/VIS spectrophotometer, and the 100X working solutions in PBS buffer were made before each titration.

The concentration of the proteins was evaluated using sample absorption at 280 nm and extinction coefficients computed by ProtParam tool (http://web.expasy.org/protparam/). To reduce dilution-induced artifacts during titration, series of diluted protein solutions were also prepared.

The fluorophore solution (10 μl) and required amount of protein solution and PBS up

to 1 ml were added into cuvette, gently stirred and immediately measured using Varian Cary Eclipse fluorescence spectrophotometer. For each fluorophore-protein pair points with at least two different fluorophore and fifteen protein concentrations were measured.

Analysis of fluorescence titration data

The fluorescence titration data were fitted to the 1:1 binding model. Supposing Blc-mutant proteins as monomers with single binding site for fluorophore, the binding of fluorophore to Blc-mutant protein can be described as P + F ⇌ PF, where P is free protein, F is free fluorophore, and PF is protein-fluorophore complex. Then the dissociation constant (K_d) equation is:

$$K_d = \frac{[P]_{eq}[F]_{eq}}{[PF]_{eq}},$$

where $[P]_{eq}$ is free protein, $[F]_{eq}$ is free fluorophore, and $[PF]_{eq}$ is protein-fluorophore concentrations at equilibrium.

As we knew only initial concentration of the protein and the fluorophore, and had not any tool for the free protein and the free fluorophore concentration at equilibrium measurement, the K_d equation was rewritten using mass balance equations:

$$[P]_{init} = [P]_{eq} + [PF]_{eq}$$

$$[F]_{init} = [F]_{eq} + [PF]_{eq}$$

where $[P]_{init}$ and $[F]_{init}$ are initial concentration of the protein and the fluorophore, respectively:

$$[P]_{eq} = [P]_{init} - [PF]_{eq}$$

$$[F]_{eq} = [F]_{init} - [PF]_{eq}$$

$$K_d = \frac{([P]_{init} - [PF]_{eq})([F]_{init} - [PF]_{eq})}{[PF]_{eq}}$$

$$([P]_{init} - [PF]_{eq})([F]_{init} - [PF]_{eq}) = K_d [PF]_{eq}$$

$$[P]_{init}[F]_{init} - [PF]_{eq}[F]_{init} - [P]_{init}[PF]_{eq} + [PF]_{eq}^2 - K_d[PF]_{eq} = 0$$

$$[PF]_{eq}^2 - [PF]_{eq}([F]_{init} + [P]_{init} + K_d) + [P]_{init}[F]_{init} = 0$$

The protein-fluorophore complex ($[PF]_{eq}$) concentration can be expressed as:

$$[PF]_{eq} = \frac{([F]_{init} + [P]_{init} + K_d) - \sqrt{([F]_{init} + [P]_{init} + K_d)^2 - 4[P]_{init}[F]_{init}}}{2} \quad \textbf{(1)}$$

Thus the fluorescence emission intensity (F) of the protein-fluorophore solution can be expressed as:

$$F = [PF]_{eq} * \Phi_{[PF]} + ([F]_{init} - [PF]_{eq}) * \Phi_{[F]} \quad \textbf{(2)}$$

where $\Phi_{[PF]}$ and $\Phi_{[F]}$ are the relative fluorescence quantum yields of the protein-fluorophore complex and the pure fluorophore, respectively.

Therefore to determine apparent K_d, the experimentally obtained data were fitted to equation 2 by *leastsq* method from Scipy *optimize* package.

Quantum yield measurement

Quantum yield of the protein-fluorophore complexes was determined by direct comparison with purified EGFP protein (quantum yield 0.6).

Supplementary references

1 T. Quan, S. Zeng and Z.-L. Huang, *J. Biomed. Opt.*, 2010, **15**, 066005.
2 M. Ovesný, P. Křížek, J. Borkovec, Z. Svindrych and G. M. Hagen, *Bioinformatics*, 2014, **30**, 2389–2390.
3 M. S. Baranov, K. M. Solntsev, K. A. Lukyanov and I. V. Yampolsky, *Chem. Commun.* , 2013, **49**, 5778–5780.
4 M. S. Baranov, I. T. Fedyakina, M. Y. Shchelkanov and I. V. Yampolsky, *Tetrahedron*, 2014, **70**, 3714–3719.
5 M. S. Baranov, K. M. Solntsev, N. S. Baleeva, A. S. Mishin, S. A. Lukyanov, K. A. Lukyanov and I. V. Yampolsky, *Chem. Eur. J.*, 2014, **20**, 13234–13241.
6 N. V. Povarova, N. G. Bozhanova, K. S. Sarkisyan, R. Gritcenko, M. S. Baranov, I. V. Yampolsky, K. A. Lukyanov and A. S. Mishin, *J. Mater. Chem.*, 2016, **4**, 3036–3040.
7 B. Webb and A. Sali, in *Current Protocols in Bioinformatics*, John Wiley & Sons, Inc., 2002.
8 G. M. Morris, R. Huey, W. Lindstrom, M. F. Sanner, R. K. Belew, D. S. Goodsell and A. J. Olson, *J. Comput. Chem.*, 2009, **30**, 2785–2791.
9 O. Trott and A. J. Olson, *J. Comput. Chem.*, 2010, **31**, 455–461.
10 V. Campanacci, D. Nurizzo, S. Spinelli, C. Valencia, M. Tegoni and C. Cambillau, *FEBS Lett.*, 2004, **562**, 183–188.
11 A. Matsumoto and T. Q. Itoh, *Biotechniques*, 2011, **51**, 55–56.
12 A. D. Edelstein, M. A. Tsuchida, N. Amodaj, H. Pinkard, R. D. Vale and N. Stuurman, *J Biol Methods*, , DOI:10.14440/jbm.2014.36.
13 E. Hoogendoorn, K. C. Crosby, D. Leyton-Puig, R. M. P. Breedijk, K. Jalink, T. W. J. Gadella and M. Postma, *Sci. Rep.*, 2014, **4**, 3854.
14 J. Schindelin, I. Arganda-Carreras, E. Frise, V. Kaynig, M. Longair, T. Pietzsch, S. Preibisch, C. Rueden, S. Saalfeld, B. Schmid, J.-Y. Tinevez, D. J. White, V. Hartenstein, K. Eliceiri, P. Tomancak and A. Cardona, *Nat. Methods*, 2012, **9**, 676–682.
